# Variation in Soil Temperature Predicts Small Seasonal Shifts in Daily Activity Patterns of a Social Subterranean Rodent

**DOI:** 10.1177/07487304251378606

**Published:** 2025-11-14

**Authors:** Kyle T. Finn, Yannick Francioli, Jack Thorley, Markus Zöttl

**Affiliations:** * Kalahari Mole-rat Project, Kalahari Research Centre, Northern Cape, South Africa; † Department of Biology and Environmental Science, Linnaeus University, Kalmar, Sweden; ‡ Department of Biology, The University of Texas at Arlington, Texas, USA; § Department of Zoology, University of Cambridge, Cambridge, UK

**Keywords:** locomotor activity, diel rhythm, Bathyergidae, biologging, chronobiology

## Abstract

Animals often show distinct activity rhythms which may align their behavior with favorable environmental conditions. In terrestrial species, daily and seasonal activity patterns are largely influenced by changes in photoperiod and temperature. However, subterranean animals experience weak or absent environmental variation due to minimal light exposure and reduced daily temperature fluctuations. Despite these conditions, many subterranean rodents display pronounced diel rhythms in physiological processes and locomotor activity, though the extent of seasonal variation remains unclear. In this study, we used radio frequency identification technology on wild groups of subterranean Damaraland mole-rats to assess their daily activity patterns. Our results show a population-wide daily activity peak around midday, which coincides with the minimum temperature at nesting depths and increasing temperature at foraging depths. The timing of this peak shifts by approximately 2 h between seasons. Neither individual nor group characteristics predicted the occurrence and timing of the activity peak, suggesting that temperature fluctuations, rather than social factors, are the main driver of seasonal variation in activity timing. Although Damaraland mole-rats remain active at low levels throughout the day, they display clear diurnal foraging rhythms at the group level that change little across seasons.

## Graphical abstract

**Graphical abstract:**
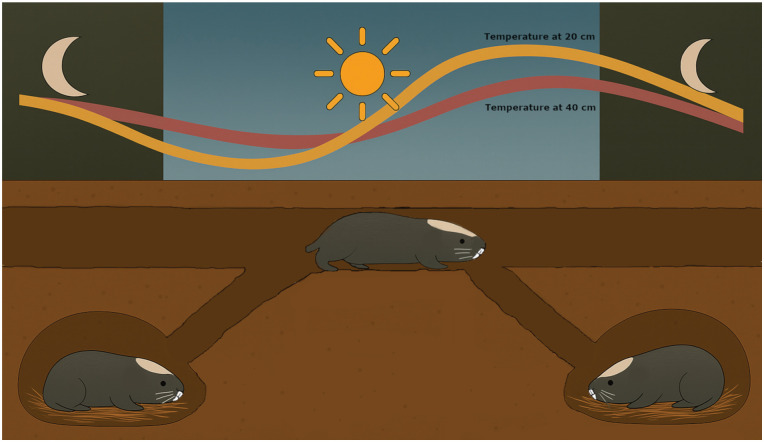
Damaraland mole-rats time their foraging bouts in shallow tunnels (20 cm below ground) to the minimum temperature at the depths of their nests (40 cm below ground). When temperatures in the foraging tunnels begin to increase they will retreat back to deeper nests.

Animals will adjust their activity rhythms with favorable environmental conditions, ultimately increasing their survival (e.g. foraging success, avoid predation) (Bartness and Albers, 2000; Getz, 2009; Yerushalmi and Green, 2009). Most animals show pronounced genetically controlled rhythmicity at different temporal scales as an adaptive survival mechanism under daily or seasonal temperature variation (Bollinger and Schibler, 2014; Huang et al., 2011; Williams et al., 2016). Some species may adjust the timing of locomotor activity to avoid temperature extremes (Hoogenboom et al., 1984; Kenagy et al., 2002; Williams et al., 2014), alleviate competition with other species (Sęk et al., 2023), or synchronize activity bouts with neighboring conspecifics (Getz, 2009; Korslund, 2006). However, other species show attenuated or undetectable rhythms over extended periods, either seasonally (e.g. such as during polar summers, Lu et al., 2010; van Oort et al., 2005) or even over their entire lifespans (Duboué et al., 2011; Kavanau, 1998). These exceptions are generally found in species living in stable environments where ecological conditions rarely fluctuate and allow for around-the-clock activity (Beale et al., 2016; Bloch et al., 2013).

The subterranean ecotope is one such environment which may lead to a lack of rhythmicity in diel activity (Beale et al., 2016). Subterranean mammals spend nearly their entire lives underground, rarely venturing out of their protected burrows. However, despite living in an environment with minimal light and small daily fluctuations in temperature, many species of subterranean rodents (families Bathyergidae, Ctenomyidae, Geomyidae, Octodontidae, Spalacidae) display distinct diel rhythms in locomotor activity (Benedix, 1994; Finn et al., 2022a, 2024; Flôres et al. 2021; Gettinger, 1984; Jannetti et al., 2025; Jarvis, 1973; Ji et al., 2018; Nevo, 1999; Oosthuizen and Bennett, 2022; Petrovskii and Novikov, 2024; Rado et al., 1993; Rezende et al., 2003; Silvério et al., 2025; Šklíba et al., 2014, 2016; Valentinuzzi et al., 2009; Vlasatá et al., 2017). Under laboratory conditions, African mole-rats will adjust the timing of their activity cycles to minor changes in temperatures (Grenfell et al., 2024; Hart et al., 2021; van Jaarsveld et al., 2019). Daily and seasonal temperature variation likely affects the timing of activity in wild subterranean rodents. In the wild, the activity bouts of many species peak mid-day to late afternoon—often coinciding with the highest temperatures—including in pocket gophers (*Thomomys bottae*, Gettinger, 1984), blind mole-rats (*Spalax ehrenbergi*, Rado et al., 1993; *Spalax galili*, Šklíba et al., 2016), tuco-tucos (*Ctenomys coludo*, Flôres et al., 2021; Silvério et al., 2025), giant root rats (*Tachyoryctes macrocephalus*, Vlasatá et al., 2017), and African mole-rats (*Heliophobius argenteocinereus*, Šklíba et al., 2007; *Fukomys anselli*, Šklíba et al., 2014; *Cryptomys pretoriae*, Finn et al., 2024). This peak can occur during either summer or winter seasons. In contrast, other studies revealed a bimodal activity pattern in summer, where activity peaks twice within a 24-h period. For example, activity peaked during early morning and early evening to avoid the hottest times of the day in plains pocket gophers (*Geomys bursarius*, Benedix, 1994), coruros (*Spalacopus cyanus*, Rezende et al., 2003), plateau zokors (*Eospalax baileyi*, Ji et al., 2018), and Natal mole-rats (*Cryptomys natalensis*, Finn et al. 2022a). In addition, some species may display ultradian rhythms, with multiple bouts of activity per day interspersed with resting periods (Finn et al., 2024; Petrovskii and Novikov, 2024; Silvério et al., 2025; Šklíba et al., 2007). Finally, still other species appear to lack a clear diel rhythm under natural conditions (Attwater’s pocket gophers *Geomys attwateri*, Cameron et al., 1988; giant mole-rats *Fukomys mechowii*, Lövy et al., 2013; Damaraland mole-rats *Fukomys damarensis*, Lovegrove, 1988).

Many of the studies of wild subterranean mammals have been conducted over short time frames, on the scale of weeks or a few months (Benedix, 1994; Finn et al., 2024; Gettinger, 1984; Lövy et al., 2013; Rado et al., 1993; Rezende et al., 2003, Šklíba et al., 2007, 2014, 2016). Few studies have been conducted over different seasons or an entire calendar year to incorporate seasonal variation in temperature and their effects on activity cycles (Finn et al., 2022a; Flôres et al., 2021; Ji et al., 2018; Rezende et al., 2003; Silvério et al., 2025; Šklíba et al., 2025). Such year-round studies have found that in some species, peak activity may shift from a single midday peak in winter to 2 peaks (morning and evening) during summer (coruros, Rezende et al., 2003; tuco-tucos, Flôres et al., 2021, Silvério et al., 2025; Natal mole-rats, Finn et al., 2022a), while other species may not experience this shift (plateau zokors, Ji et al., 2018). In addition, many studies have focused on solitary dwelling subterranean mammals (Benedix, 1994; Gettinger, 1984; Flôres et al., 2021; Ji et al., 2018; Rado et al., 1993; Silvério et al., 2025; Šklíba et al., 2007, 2016; Vlasatá et al., 2017), with few studies on the family-dwelling species, such as the coruros of South America (Rezende et al., 2003) and the African mole-rats (Finn et al., 2022a, 2024; Lövy et al., 2013; Šklíba et al., 2014). Social factors have been suggested to be an important contributor to timing of activity patterns in both subterranean rodents (Rezende et al., 2003; Šklíba et al., 2014) and other social organisms in general (Bloch et al., 2013; Costa Petrillo et al., 2024; Korslund, 2006). Thus, it is still unclear how social cues and seasonal temperature fluctuations throughout the year may interact to affect daily activity patterns.

In this study, we assessed the daily activity patterns of wild Damaraland mole-rats in the Kalahari region of South Africa over an entire calendar year using a biologging approach. We deployed a radio frequency identification (RFID) array consisting of a remote antenna positioned above shallow, active foraging tunnels (Francioli et al., 2020). The foraging tunnels of Damaraland mole-rats are between 20 and 40 cm below ground, which mole-rats excavate to access the underground tubers of various plant species (Bennett and Faulkes, 2000). By recording the presence of individuals in these tunnels, we were able to estimate activity. When they are not foraging, mole-rats typically rest in one or more deeper-lying nests distributed throughout their tunnel systems. We monitored 19 groups throughout the year and used an in situ soil probe to measure the soil temperature at depths coinciding with foraging and nesting areas of their burrow systems. This approach allowed us to investigate whether wild mole-rat groups display a diel rhythm in their activity patterns, and to assess how the timing and amplitude of this rhythm is affected by seasonal variations in temperature, as well as social cues (e.g. group size) and life history conditions (e.g. sex, reproductive status, body mass).

## Methods

The Damaraland mole-rat is a small (15-20 cm total length) cooperatively breeding social rodent living in the arid regions of Namibia, Botswana, and South Africa. Individuals live in extended family groups of up to 41 individuals (mean group size is 8-9 in most populations), where reproduction is monopolized by a male-female pair (Bennett and Faulkes, 2000; Thorley et al., 2023). Non-reproductive individuals engage in cooperative foraging behavior by expanding the burrow system in the search for roots and tubers (Bennett and Faulkes, 2000).

We carried out our study at the Kalahari Research Centre in the Northern Cape of South Africa (26°58’S, 21°49’E) between January 2014 and September 2015. The site experiences cold, dry winters from April to September (mean daily minimum temperature 3.1 °C) and hot summers with sporadic rainfall from October to April (mean daily maximum temperature 34.0 °C, Francioli et al., 2020; Thorley et al., 2023). Mole-rats at our study site have been monitored since October 2013, with all groups recaptured approximately every 6 months (Thorley et al., 2023). All individuals were implanted with a subcutaneous passive integrated transponder microchip (Trovan, DorsetID, The Netherlands; hereafter “transponder”) for unique identification. We sexed all individuals by visual inspections of their genitals and weighed them to the nearest gram at each capture. We identified breeding females by their perforated vagina and prominent teats (Bennett and Faulkes, 2000). Breeding males were identified through longitudinal capture records because these are usually the only large males remaining in breeding groups for over 2 years (Torrents-Ticó et al., 2018; Thorley et al., 2023). Males were heavier than females, and reproductive individuals were heavier than non-reproductive individuals (reproductive male body mass (mean + SD) = 152.5 ± 16.5 g; reproductive females = 132.2 ± 18.0 g; non-reproductive males = 106.5 ± 34.4 g; non-reproductive females = 96.0 ± 28.0 g).

### Data Collection

To detect daily activity, we used a RFID reader array over the shallow foraging tunnels of wild mole-rat groups. These tunnels can be readily identified by lines of fresh molehills formed from sand excavated during the digging process. The RFID reader array consists of a stationary decoder (LID650/608, DorsetID, The Netherlands) connected to a panel antenna (Trovan ANT 612, 47.5 × 40 × 4 cm). The array is powered by a 12 V battery and recharged with a solar panel. The panel antenna records all transponders which pass within 30 cm (see Francioli et al., 2020, for a detailed technical description). We dug through the soil to find an active tunnel and placed the antenna directly on the tunnel. Thus, detection can be over 30 cm below ground since the detection panel is not at the soil surface. We left the reader array over a group for at least 24 h, but up to 5 days in some cases (mean duration ± SD = 41.8 ± 26.2 h). Before placing the array over the group’s burrow, we captured and implanted new group members with a transponder.

We placed the RFID array repeatedly at 19 different groups (mean group size ± SD = 9.5 ± 4.5, range, 1-18) during the winter season (May-September) and again during the summer season (October-April) for a total of 39 different reading sessions. Groups were resampled a mean (± SD) of 2.1 ± 1.5 times. A total of 126 unique individuals (males = 61, females = 65) were detected by the array during the study. Of these, 11 females and 14 males were considered the group’s breeding individuals, with the remainder being non-reproductive individuals. When recapturing the groups after removing the reader, 80.4 ± 23.8% of detected group members were recaptured.

To assess how daily and seasonal variation in soil temperature may affect activity patterns, we measured the temperature at 20, 40, and 60 cm below ground using an on-site soil probe (DFM Technologies, South Africa) buried 2 m into the sand.

### Statistical Analysis

To investigate the daily activity of Damaraland mole-rats, we coded an individual as active (activity = 1) during a given hour if it was recorded at least once within that hour, and inactive (activity = 0) if it was not detected during that hour (see Francioli et al., 2020; Finn et al., 2022a). We used a binary activity score for 2 reasons. First, because our RFID panel scanned for transponders every 3 s, the distribution of detections among individuals was uneven (i.e. right skewed). Second, our analysis focused on population-level activity cycles instead of the amplitude of activity per individual. To avoid any lingering behavioral effect of placing the RFID array (i.e. increased activity to repair the tunnel after placing the panel), we removed the first 4 h of data. This resulted in a sample size of 2258 hourly detections across all groups.

We separated our analysis into 4 different aspects: (1) daily variation (cosine and sine wave of radian time), (2) individual characteristics (sex, body mass, breeding status) and social characteristics (group size), (3) seasonal variation (cosine and sine wave of the radian date), and (4) temperature variation at different depths (20, 40, and 60 cm). For each of those aspects, we fitted separate binomial generalized linear mixed models (GLMM) with activity (0/1) as the response variable and various predictor variables and/or their interactions. We assessed each model’s predictive power using the Akaike information criterion (AIC, Burnham and Anderson, 2002). All models also included the time passed since the start of the reading session as a fixed effect, along with individual identity and reading session as random effects. Significance was determined using α = 0.05. Means are given as mean + SD.

#### Variation in Diel Activity Patterns

To assess whether mole-rats display a diel rhythm in activity patterns—characterized by one or multiple peaks of activity over a 24-h period—we built 4 separate GLMMs. Each model included sine and cosine terms representing periodicity with 1 to 4 cycles per day (Stolwijk et al., 1999). We first converted the time of day to radians and fitted radian time to a cosine and a sine function, so that for a monophasic rhythm the hour spanned 0 to 2π, a biphasic rhythm spanned 0 to 4π, and so on. The 4 cosine and sine waves were then used as fixed predictors of daily activity in 4 separate GLMMs to determine how many phases (i.e. 1 to 4) of activity mole-rats are likely to have in a 24-h period. While individual mole-rats may show multiple bouts of activity throughout the day, this analysis identifies the predominant times of day when activity is most likely to occur at the population level.

#### Individual and Group Characteristics

The sets of models to test for the effect of individual and social characteristics also incorporated the radian time in addition to other fixed effects. The individual characteristics model included sex, reproductive status, and body mass as fixed effects. The model assessing social effects on activity included only group size at last capture as a fixed effect. Both models included the cosine and sine wave as covariates of the fixed effects, which accounts for hourly variation in activity.

#### Seasonal Variation

To predict seasonal variation in activity, we used the same process as daily variation. We converted the date to a value between 0 and 2π, so that January 1st is 0 and December 31st is 2π. Then both radian time and radian date were used as covariates to predict seasonal changes in daily activity patterns.

#### Temperature Effects on Activity

To investigate the relation between activity and temperature variation at different depths, we compared 3 separate models using soil temperature variation at 20, 40, and 60 cm as predictors for activity. These depths were selected because they coincide with the depth of foraging tunnels (Bennett and Faulkes, 2000). Temperature variation was obtained by subtracting the mean daily temperature from the hourly temperature records (i.e. within-day variation). Because mole-rats retreat to a deep nest during inactive periods (Šklíba et al., 2007, 2014), we assume that if active the mole-rats were moving in relatively shallow tunnels (20-40 cm below ground).

## Results

### Diel Activity and the Effects of Individual Characteristics

**Figure 1.**
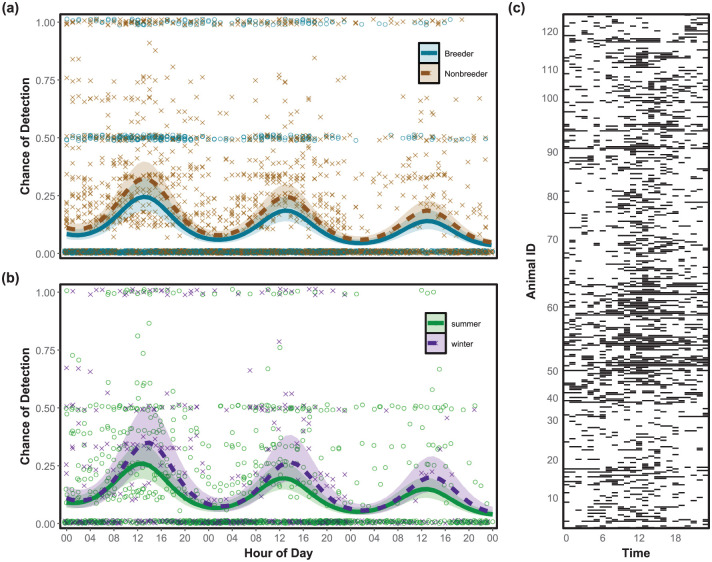
Variation in diel activity of Damaraland mole-rats over 3 days. (a) The predicted activity for breeders (blue solid line) and non-breeders (brown dashed line). The crosses show the proportion of non-reproductive mole-rats active in a group across all reading sessions (*n* = 39). The open circles indicate if neither of the 2 reproductive individuals were active (values 0), at least one was active (values 0.5), or both were active (values 1). (b) The predicted activity during the hottest summer months (December-February, green solid line) and coldest winter months (June-August, purple dashed line). The crosses represent the proportion of within-group activity per hour for groups sampled during winter (*n* = 8), and the circles for groups in summer (*n* = 15). Activity was detected using a RFID reader array placed above active tunnels. The likelihood of activity was predicted using a generalized linear model (GLMM) with time of day (in radians) and either reproductive status or month (in radians), respectively, as the response variables. The shaded regions show the 95% CI. (c) Actogram of all 126 detected mole-rats. Black bars indicate at least one detection under the RFID array within that hour.

**Figure 2.**
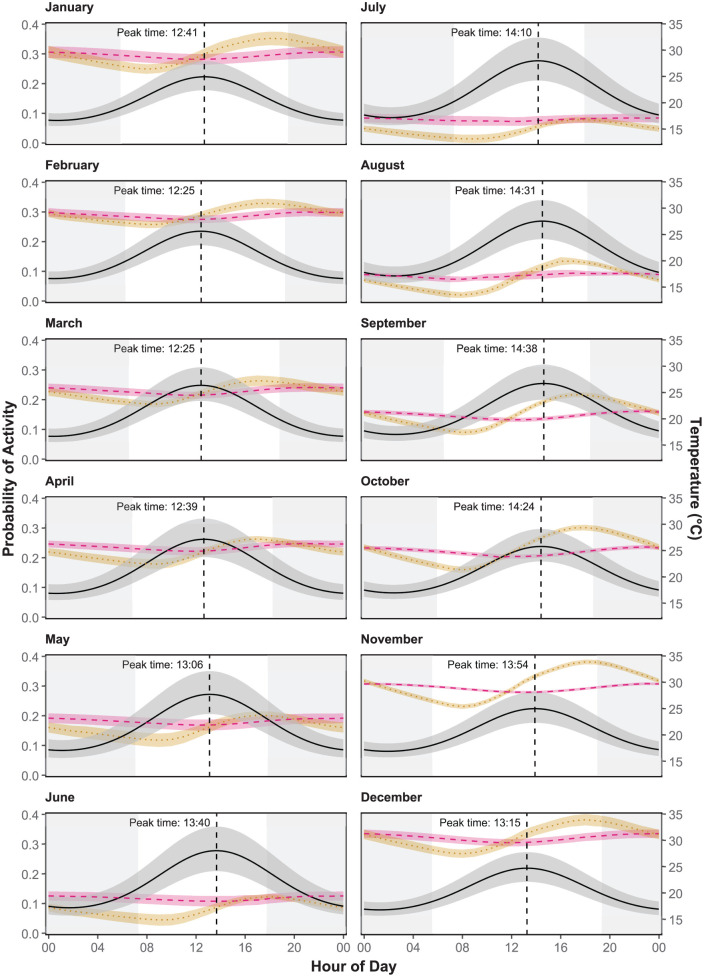
Change in diel activity across seasons. The black horizontal line shows the predicted activity of mole-rats from a generalized linear model using the month to predict likelihood of activity. The vertical line represents the time of the peak in daily activity. The mean daily variation in soil temperature is shown for 20 cm below ground (orange dotted line) and 40 cm (red dashed line). Confidence intervals are represented in the shaded regions. White background indicates period of daylight.

The diel activity patterns of Damaraland mole-rat groups were best explained by the monophasic model, which predicted a single pronounced peak of daily group level activity between 1200 and 1400 h (Figures 1 and 2, Table 1). The model with 2 activity cycles per 24 h did not explain the activity patterns better than a monophasic model (Table 1, Supplemental Figure S1).

**Table 1.** Number of activity phases per day in Damaraland mole-rats.

	Intercept	Cos(hour)	Sin(hour)	Time From Start	AIC	ΔAIC	logLik	W	df
*1-phasic model*									
Estimate	-1.448	-0.592	-0.236	-0.012	11437.5	0	-5712.8	1	6
Std Error	0.098	0.035	0.035	0.001					
*p*-value	**<0.001**	**<0.001**	**<0.001**	**<0.001**					
*2-phasic model*									
Estimate	-1.476	0.184	0.008	-0.009	11751.4	313.9	-5869.7	0	6
Std Error	0.094	0.034	0.034	0.001					
*p*-value	**<0.001**	**<0.001**	0.807	**<0.001**					
*3-phasic model*									
Estimate	-1.459	-0.026	-0.066	-0.009	11777.3	339.8	-5882.6	0	6
Std Error	0.094	0.033	0.034	0.001					
*p*-value	**<0.001**	0.435	0.048	**<0.001**					
*4-phasic model*									
Estimate	-1.463	-0.009	-0.003	-0.009	11781.7	344.2	-5884.9	0	6
Std Error	0.093	0.033	0.034	0.001					
*p*-value	**<0.001**	0.788	0.930	**<0.001**					

To predict the most likely number of activity phases within a 24-h period, we fitted a cosine-sine wave to activity values in a generalized linear model (GLMM). Each model tested for a different number of activity phases per day (1-4 phases). Individuals were scored as active (Activity = 1) if they were recorded passing under a radio frequency identification (RFID) panel at least once within a given hour. Individuals were scored as inactive (Activity = 0) if they were not detected during that hour. Time of day was converted to radians. Time From Start is the amount of time that passed since the beginning of the reading session. Significant values are in bold. AIC = Akaike information criterion; ΔAIC = change in AIC from the best supported model; logLik = log-likelihood; W = Akaike’s model weight; df = degrees of freedom.

Within groups, reproductive individuals of both sexes showed reduced activity compared to non-reproductive individuals (Table 2, Figure 1a). The amount of activity may change for breeders and non-breeders depending on the season, with non-breeders increasing their activity levels during winter and breeders decreasing their activity (Supplemental Figure S2). However, only one of these interactions was significant (Supplemental Table S1). The activity levels were not affected by group size, sex, or body mass (Table 2). These results suggested that, conditional on their reproductive status, individuals from all groups in the study spent similar proportions of time active in foraging tunnels despite living in groups of varying sizes.

**Table 2.** Effects of life history characteristics and group size on diel activity patterns.

	Estimate	Std Error	*p*-value
*Life History Characteristics*			
Intercept	-1.627	0.114	**<0.001**
Time-from-Start	-0.012	0.001	**<0.001**
Sex	0.128	0.103	0.214
Body mass	0.002	0.001	0.267
Breeding status	-0.349	0.142	**0.013**
Cos(hour)	-0.593	0.035	**<0.001**
Sin(hour)	-0.236	0.035	**<0.001**
*Group Size*			
Intercept	-1.131	0.222	**<0.001**
Time-from-Start	-0.012	0.001	**<0.001**
Group size	-0.032	0.020	0.112
Cos(hour)	-0.592	0.035	**<0.001**
Sin(hour)	-0.236	0.035	**<0.001**

Results are from GLMMs using a cosine-sine wave on hourly activity scores. Individuals were scored as active (Activity = 1) if they were recorded passing under a radio frequency identification (RFID) panel at least once within a given hour. Individuals were scored as inactive (Activity = 0) if they were not detected during that hour. Time-from-Start is the amount of time that passed since the beginning of the reading session. Significant values are in bold.

Further analysis of the interactions between individual variables (sex, body mass, breeding status) and the hour of the day showed no significant effects (Supplemental Table S2). These results imply that these variables do not affect the timing of the peak of activity. However, one interaction of group size and hour of the day was significant (Supplemental Table S2). This result may indicate that the peak of activity for smaller groups may be up to 2 h later than the peak for larger groups (Supplemental Figure S3).

### Activity and the Effects of Temperature Variation

The temperature variation at 40 cm below ground best predicted diel activity compared to temperatures at 20 and 60 cm (Table 3). The peak of daily activity varied slightly between seasons, shifting by 2 h over the year (Figure 1b, Table 4). The peak of detection in shallow foraging tunnels coincided significantly with the lowest temperature at 40 cm between 1100 and 1300 h (Supplemental Table S3, Supplemental Figure S4), as well as increasing temperatures at 20 cm (Figure 2). During the hot summer months (December-February), mole-rat activity peaked between 1200 and 1230 h (Figure 2). At the onset of winter in June, the activity peak began to delay, moving to around 1410 h during the coldest month, before advancing again in late spring and early summer (Figure 2). See Supplemental Figure S5 for an enlarged figure which better shows the daily variation in temperature at 40 cm for each month.

**Table 3.** Temperature effects on the timing of diel activity patterns.

	Temperature variation	Time From Start	AIC	ΔAIC	logLik	W	df
*Soil temperature variation at 40* *cm*							
Estimate	0.198	-0.009	11704.0	0	-5847.0	1	5
Std Error	0.023	0.001					
*p*-value	**<0.001**	**<0.001**					
*Soil temperature variation at 60* *cm*							
Estimate	-0.027	-0.009	11779.3	75.3	-5884.7	0	5
Std Error	0.04	0.001					
*p*-value	0.49	**<0.001**					
*Soil temperature variation at 20* *cm*							
Estimate	0.018	-0.009	11775.4	71.4	-5882.7	0	5
Std Error	0.009	0.001					
*p*-value	0.037	**<0.001**					

Results are from separate GLMMs comparing how activity (binary; 1 = Active, 0 = Inactive) is affected by soil temperature at 20 cm, 40 cm, and 60 cm. Significant values are in bold. AIC = Akaike information criterion; ΔAIC = change in AIC from the best model; logLik = log-likelihood; W = Akaike’s model weight; df = degrees of freedom.

**Table 4.** Seasonal effect on timing of activity patterns.

	Estimate	Std Error	*p*-value
Intercept	-1.431	0.101	**<0.001**
Sin(hour)	-0.246	0.037	**<0.001**
Cos(hour)	-0.601	0.037	**<0.001**
Sin(year)	< 0.001	0.119	1.000
Cos(year)	-0.107	0.130	0.411
Time-from-Start	-0.012	0.001	**<0.001**
Sin(hour)*Cos(year)	0.097	0.052	0.059
Sin(hour)*Sin(year)	0.152	0.048	**0.001**
Cos(hour)*Cos(year)	0.014	0.053	0.794
Cos(hour)*Sin(year)	-0.094	0.049	0.056

Results are from a GLMM using a cosine-sine wave to predict annual variation in the peak of activity. Seasonal activity variation was modeled as cosine-sine(year) and hourly variation as cosine/sine(hour). The hour and calendar day of activity were converted to radians. Individuals were scored as active (Activity = 1) if they were recorded passing under a radio frequency identification (RFID) panel at least once within a given hour. Individuals were scored as inactive (Activity = 0) if they were not detected during that hour. Significant values are in bold.

The soil temperatures at 20 cm showed the greatest daily variation (4-8 °C change), while temperatures at depths over 60 cm exhibited minimal daily variation (often < 1 °C, Supplemental Table S4). The variation in daily temperature at 20 cm was greatest from winter through to early spring (August-December, Supplemental Table S4). The variation in daily temperature at 40 cm was greatest during early autumn (August) and summer (December and February) (Supplemental Table S4). The annual mean temperature at 40 cm reached its maximum in December (30.4 ± 4.48 °C) and minimum in July (16.8 ± 3.84 °C, Supplemental Table S5).

## Discussion

Laboratory studies of activity in African mole-rats indicate that individual activity patterns vary across 24 h, with some individuals being nocturnal and others diurnal; some having a single peak in activity and others with 2 peaks of activity, and still others with no clear rhythm (arrhythmic) (de Vries et al., 2008; Grenfell et al., 2024; Lovegrove et al., 1993; Oosthuizen et al., 2003; Oosthuizen and Bennett, 2015; Riccio and Goldman, 2000; van Jaarsveld et al., 2019). Our results on wild Damaraland mole-rats suggest a primarily diurnal diel rhythm with a single peak between 1200 and 1400 h. This time period coincided with the minimum daily temperature at 40 cm below ground (Figure 2). Even though soil temperature at this depth varied relatively little over the course of a day, there was substantial variation across the year (Figure 2, Supplemental Tables S4 and S5). This result is similar to a laboratory study where Damaraland mole-rats kept in constant darkness and with a natural temperature cycle were more active at the lowest temperatures (18-23 °C, Grenfell et al., 2024). Our data also show that many individuals were active throughout a 24-h period (Figure 1c). The timing of the peak varies little, albeit significantly, across seasons. In addition, our results show that the probability of activity (i.e. amplitude of the curve) increases with decreasing burrow temperatures during the dry winter season (Figure 2). This result is in contrast to a study on silvery mole-rats (*Heliophobius argenteocinereus*), where out of nest activity decreased during the dry season (Šklíba et al., 2007). As Damaraland mole-rats did not shift their activity peak substantially between seasons, their activity rhythms more closely resemble those of tuco-tucos (*Ctenomys coludo*, Silvério et al., 2025) compared to patterns found in other African mole-rat species (Finn et al., 2022a; Šklíba et al., 2025). As with our previous work on this population (Francioli et al., 2020; Rotics et al., 2025), we found that reproductive individuals exhibit decreased activity compared to subordinate individuals (Figure 1a).

Our results indicate that Damaraland mole-rats exhibit a peak of activity mid-day to early afternoon to coincide with the minimum daily temperatures at 40 cm below ground. Their decrease in daily activity also coincided with the increase in temperatures at 20 cm. These depths are where the majority of their food resources can be found. Because our study design placed the RFID array adjacent to newly constructed tunnels, we suggest that foraging, digging, or other physiologically intense (i.e. heat generating) behaviors may be focused during the time of lowest burrow temperatures, potentially to minimize overheating. Mole-rats may spend little time foraging and digging outside these times. A recent study on wild tuco-tucos using accelerometers has also shown that digging and locomotion behaviors primarily occurs during the day, while resting occurs at night (Jannetti et al., 2025). Wild mole-rats will retreat to a nest chamber at the end of activity bouts and may spend over 60% of the day resting in a nest (Šklíba et al., 2007; Lövy et al., 2013). The nest chambers of Damaraland mole-rats are typically 30-60 cm belowground, but possibly at much deeper depths of 2 m or more (Bennett et al., 1988; Lovegrove and Painting, 1987; Thomas et al., 2016). While it is unknown how much time mole-rats spend at deeper depths, they must forage at shallower depths and time spent at deeper tunnels may only be during transit to shallow tunnels. However, it has been suggested that mole-rats may retreat to deeper tunnels—which have minimal daily temperature variation—as a behavioral thermoregulation mechanism to avoid temperature extremes (Bennett et al., 1988; Lövy et al., 2013; Šklíba et al., 2014).

Some studies in other subterranean mammals have found that individuals may avoid activity during the hottest period of the day, sometimes resulting in bimodal activity peaks (Benedix, 1994; Finn et al., 2022a; Lövy et al., 2013). For example, the Natal mole-rat was found to be most active mid-day during winter, but avoided mid-day activity during summer (Finn et al., 2022a). While both Damaraland (this study) and Natal mole-rats (Finn et al., 2022a) live in environments which experience winter air temperatures below freezing, summer air temperatures are much higher in the Kalahari. Of the wild species of mole-rats studied to date (silvery, giant, highveld, and Natal), Damaraland mole-rats have the deepest foraging tunnels (30-40 cm) compared to the other species (5-10 cm). The deeper foraging tunnels of Damaraland mole-rats may provide a greater thermal buffer against daily temperature variation compared to species with shallower tunnels. In addition, species like Ansell’s mole-rat and silvery mole-rat may live in forested habitats (Šklíba et al., 2007, 2014) where shade may provide a cooling effect to the soil. This cooling effect may shift the duration of optimal temperature later in the day, and activity in temperatures outside their thermal neutral zone may be costly. Therefore, mole-rats may time their activity cycles to the optimal daily temperature (either the minimum or maximum) to prevent expending energy to maintain a stable body temperature. These relationships indicate that burrow depth alone may not predict the timing of activity peaks.

The monophasic pattern observed in this study raises the question of which *zeitgeber* is used by wild mole-rats to entrain their endogenous circadian clock, light, or temperature. In laboratory studies, light is the most frequently tested *zeitgeber* in African mole-rats (Oosthuizen and Bennett, 2022), but daily temperature variation can also affect activity cycles (Goldman et al., 1997; Grenfell et al., 2024; Hart et al., 2021; Haupt et al., 2017; van Jaarsveld et al., 2019). In the absence of light, mole-rats can entrain their endogenous rhythms to temperature cycles in captivity and will adjust their cycles after a phase shift in temperature (Grenfell et al., 2024; van Jaarsveld et al., 2019). Even infrequent light pulses which mimic the sporadic light exposure during mound creation was enough to entrain the circadian clock in some subterranean rodents (Flôres et al., 2016; Rado et al., 1993). While temperature cycles serve as a *zeitgeber* for some mammals in the absence of light (Farsi et al., 2020a, 2020b), it is unclear whether temperature acts as a *zeitgeber* or a masking factor in African mole-rats. It is unlikely that wild Damaraland mole-rats are exposed to light while living in their subterranean burrows. Most mole-rats do not perform surface forays and may travel on the surface only once in their life during dispersal (Finn et al., 2022b). The other means of exposure to light is during mound construction. For Damaraland mole-rats, excess sand from tunnel construction is usually pushed through from below without creating an open hole to the surface. Furthermore, the daily peak of activity coincided with the lowest temperature at 40 cm, the foraging depth of their tunnels. These pieces of evidence suggest that light is unlikely to be a *zeitgeber* in wild Damaraland mole-rats.

It has been suggested that social cues may affect the timing of activity patterns in group dwelling species (Bloch et al., 2013; Costa Petrillo et al., 2024; Favreau et al., 2009). Early work in humans and bats showed that the timing of activity rhythms can be synchronized through social cues despite living in complete darkness (Aschoff et al., 1971; Marimuthu et al., 1981). In eusocial honey bees, social synchronization of activity ensures group efficiency and increases fitness (Bloch et al., 2013). When individual bees were removed from the group their timing of activity drifted from the colony’s timing. Circadian rhythm experiments in captive social bathyergids have found similar results. Isolated individuals from the same group, but exposed to the same lighting regime, may alter their circadian rhythms, desynchronizing from other group members (Grenfell et al., 2024; Oosthuizen and Bennett, 2022). In addition, a wild female giant mole-rat which self-isolated in a burrow showed a peak of activity coinciding with the peak in daily burrow temperatures, yet the group exhibited more nocturnal behavior (Lövy et al., 2013). Our results found that the overall amount of activity was consistent across groups of various sizes (used as a proxy for social cues). This result is surprising because theory predicts that a larger workforce would reduce the amount of overall group effort in digging and foraging activities. We would have expected larger groups to exhibit decreased activity compared to smaller groups due to the effort of maintaining tunnels being distributed over more individuals. Our study design prevented us from rigorously testing the effects of social cues on activity rhythms. First, the RFID panel does not collect continuous activity data. Second, our sample size of detections across different group sizes was not evenly distributed. Therefore, our results do not provide any conclusive evidence for or against group level synchronization in social subterranean rodents. To fully understand how social cues may entrain circadian rhythms in mole-rats and lead to the synchronization of activity bouts, continuous data collection using accelerometer devices would be required (*sensu* Jannetti et al., 2025; Rotics et al., 2025; Silvério et al., 2025).

## Supplemental Material

sj-pdf-1-jbr-10.1177_07487304251378606 – Supplemental material for Variation in Soil Temperature Predicts Small Seasonal Shifts in Daily Activity Patterns of a Social Subterranean Rodent Supplemental material, sj-pdf-1-jbr-10.1177_07487304251378606 for Variation in Soil Temperature Predicts Small Seasonal Shifts in Daily Activity Patterns of a Social Subterranean Rodent by Kyle T. Finn, Yannick Francioli, Jack Thorley and Markus Zöttl in Journal of Biological Rhythms
