# Supplementary material for: Variation in Soil Temperature Predicts Small Seasonal Shifts in Daily Activity Patterns of a Social Subterranean Rodent
Source: J Biol Rhythms. 2025 Nov 14;41(1):68–80. doi: 10.1177/07487304251378606 (PMC12804433; doi:10.1177/07487304251378606)
Supplement: sj-pdf-1-jbr-10.1177_07487304251378606 – Supplemental material for Variation in Soil Temperature Predicts Small Seasonal Shifts in Daily Activity Patterns of a Social Subterranean Rodent [file sj-pdf-1-jbr-10.1177_07487304251378606.pdf]

Supplementary Information for 'Daily activity rhythms vary between seasons in a wild population of social Damaraland mole-rats (*Fukomys damarensis*)'

Kyle T. Finn, Yannick Francioli, Jack Thorley, Markus Zöttl

Corresponding author email: kyletfinn@gmail.com

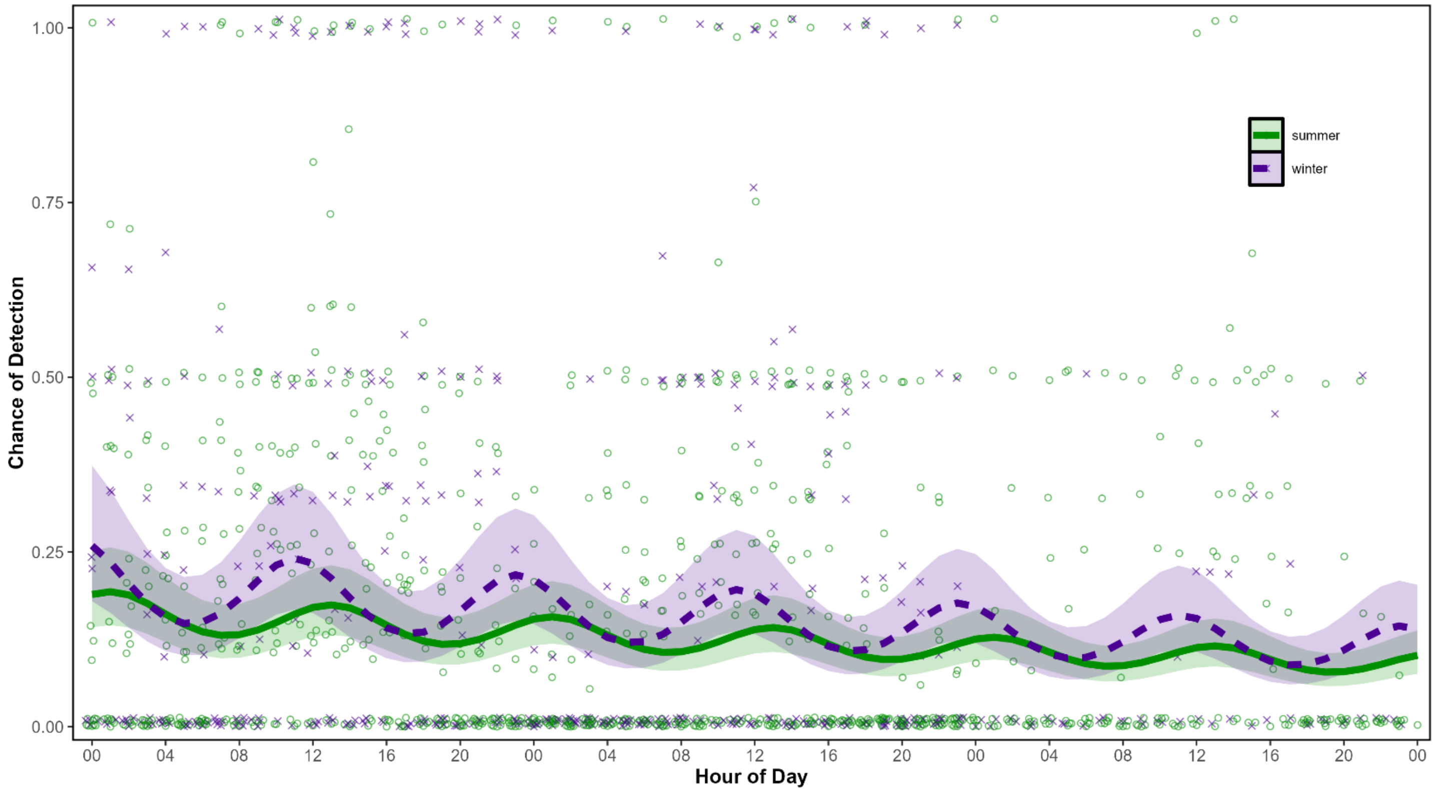

Supplemental Figure S1: Variation in diel activity over 3 days during summer (December – February, green solid line) and winter (June – August, purple dashed line) assuming 2 peaks of activity per 24-hours. The likelihood of activity was predicted using a generalized linear model (GLMM) using time of day and month as the response variables. The 95% CI is shown as the shaded region. The crosses show the proportion of mole-rats active in a group at a given hour of the day for reading sessions during summer (n = 15) and winter (*n* = 8).

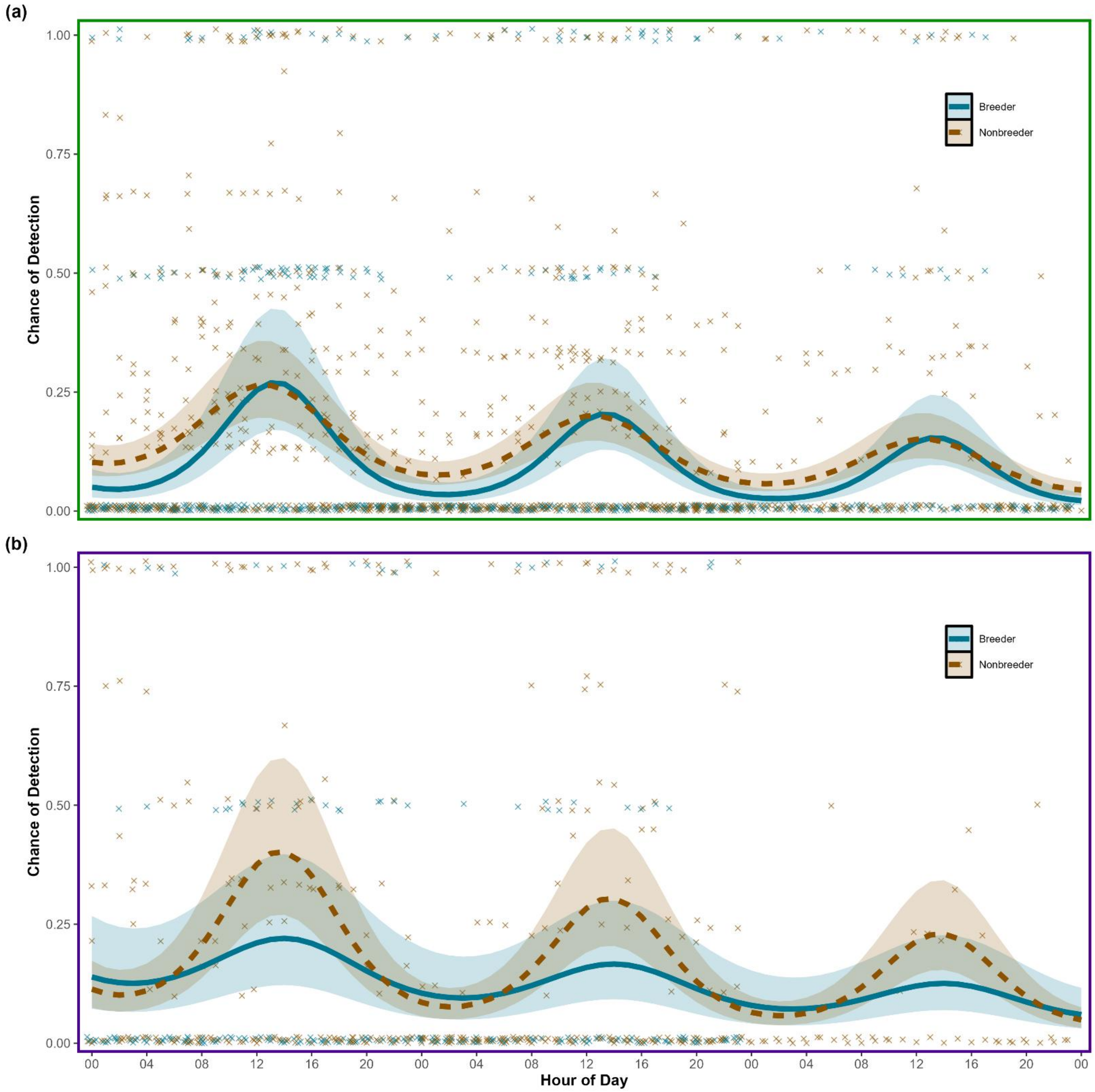

Supplemental Figure S2: Variation in diel activity of Damaraland mole-rats over 3 days between seasons for breeders (solid line) and non-breeders (dashed line) during A) the hottest summer months (December – February, green outline) and B) during the coldest winter months (June – August, purple outline). The likelihood of activity was predicted using a generalized linear model (GLMM) using time of day (in radians) and month of the season (in radians, summer = 0, winter = π) as the response variables. This model indicated there may be a shift in activity patterns for individuals of different reproductive classes between the seasons. Breeders may increase their activity during summer, while non-breeders may increase activity during winter. However, only one time/season and breeder interaction term (out of 8 total) was significant (see Supplemental Table S1 below). The 95% CI is shown as the shaded region. The crosses show the proportion of breeding and non-breeding mole-rats active in a group at a given hour of the day.

Supplemental Table S1: Effects of interactions between season and reproductive status on diel activity patterns. Results are from a GLMM using a cosine-sine wave on hourly activity scores including the interactions between time of day, time of year, and reproductive status. This interaction represents how the peak of activity can change between seasons for reproductive and non-reproductive individuals. Time and month were both converted to radians. Animals were scored as Active or Inactive if they were detected under a RFID panel in each hour. Time From Start is the amount of time that passed since the beginning of the reading session.

**Predictors**	**Est.**	**S.E.**	**p-value**
(Intercept)	-1.371	0.106	**< 0.001**
sin(hour)	-0.240	0.040	**< 0.001**
cos(hour)	-0.607	0.040	**< 0.001**
sin(year)	-0.037	0.124	0.765
cos(year)	-0.114	0.137	0.406
Breeding Status	-0.282	0.139	**0.043**
Time-from-Start	-0.012	0.001	**< 0.001**
sin(hour)*sin(season)	0.151	0.052	**0.004**
sin(hour)*cos(season)	0.124	0.056	**0.026**
cos(hour)*cos(season)	0.062	0.057	0.278
cos(hour)*sin(season)	-0.100	0.053	0.064
sin(hour)*Breeding Status	-0.055	0.098	0.575
cos(hour)* Breeding Status	0.026	0.100	0.795
sin(year)* Breeding Status	0.199	0.129	0.122
cos(year)* Breeding Status	-0.095	0.153	0.535
sin(hour)*sin(season)* Breeding Status	0.050	0.136	0.712
sin(hour)*cos(season)* Breeding Status	-0.209	0.152	0.167
cos(hour)*cos(season)* Breeding Status	-0.365	0.156	**0.019**
cos(hour)*sin(season)* Breeding Status	0.115	0.139	0.407

Supplementary Table S2: Effects of interactions of life history characteristics or group size on diel activity patterns. Results are from GLMMs using a cosine-sine wave on hourly activity scores including an interaction between time and one of the following variables: sex, body mass, reproductive status, and group size. These interactions represent how the peak of activity can change between sexes, between individuals of differing size or reproductive status, or between groups with different group sizes. Time of day was converted to radians. Individuals were scored as active (Activity = 1) if they were recorded passing under a radio frequency identification (RFID) panel at least once within a given hour. Individuals were scored as inactive (Activity = 0) if they were not detected during that hour. Time From Start is the amount of time that passed since the beginning of the reading session. Empty columns indicate terms not included in the models.

	**Time – Sex Interaction**			**Time – Body Mass Interaction**			**Time – Reproductive Status Interaction**			**Time – Group Size Interaction**		
Predictors	**Est.**	**SE**	**p-value**	**Est.**	**SE**	**p-value**	**Est.**	**SE**	**p-value**	**Est.**	**SE**	**p-value**
(Intercept)	-1.629	0.189	**< 0.001**	-1.602	0.190	**< 0.001**	-1.626	0.189	**< 0.001**	-1.160	0.223	**< 0.001**
sin(hour)	-0.275	0.049	**< 0.001**	-0.289	0.120	**0.016**	-0.221	0.038	**< 0.001**	-0.517	0.093	**< 0.001**
cos(hour)	-0.592	0.050	**< 0.001**	-0.421	0.122	**< 0.001**	-0.589	0.038	**< 0.001**	-0.694	0.095	**< 0.001**
Sex	0.131	0.103	0.206	0.127	0.103	0.216	0.128	0.103	0.213			
Breeding Status	-0.350	0.141	**0.013**	-0.352	0.141	**0.013**	-0.360	0.143	**0.012**			
Body Mass	0.002	0.001	0.267	0.001	0.001	0.338	0.002	0.001	0.265			
Group Size										-0.030	0.020	0.144
Time-from-start	-0.012	0.001	**< 0.001**	-0.012	0.001	**< 0.001**	-0.012	0.001	**< 0.001**	-0.012	0.001	**< 0.001**
sin(hour)*Sex	0.077	0.068	0.262									
cos(hour)*Sex	-0.002	0.070	0.978									
sin(hour)*Body Mass				< 0.001	0.001	0.648						
cos(hour)*Body Mass				-0.002	0.001	0.140						
sin(hour)*Breeding Status							-0.098	0.095	0.301			
cos(hour)*Breeding Status							-0.021	0.097	0.825			
sin(hour)*Group Size										0.025	0.008	**0.001**
cos(hour)*Group Size										0.009	0.009	0.252

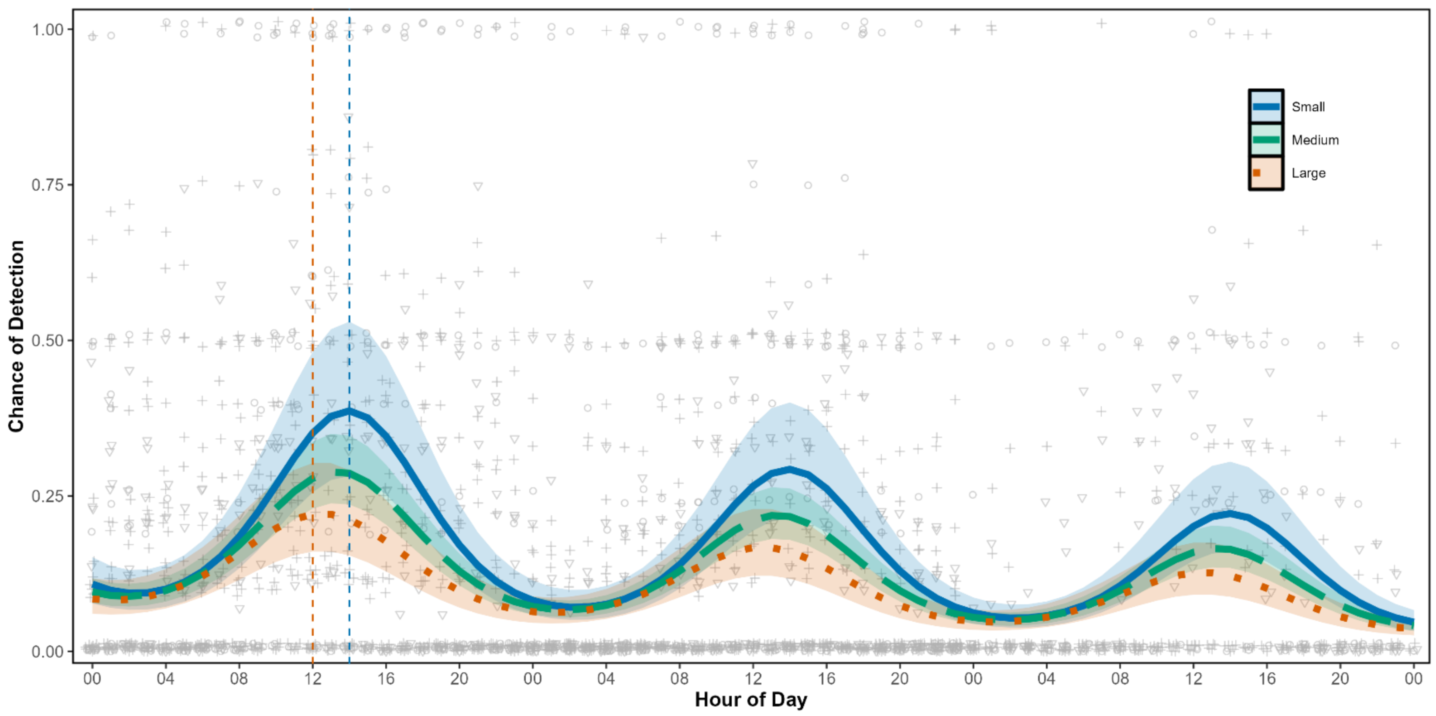

Supplemental Figure S3: Group size effects on the peak of daily activity. Curves represent the activity for individuals in small (blue solid line), medium (green dashed line), and large groups (red dotted line). The likelihood of activity was predicted with a GLMM using time of day (in radians) and group size (continuous variable) as the response variables (see Supplemental Table S2 above). The results indicate that individuals in small groups may be active up to 2 hours after individuals in larger groups. Activity was predicted using a GLMM using time of day (in radians) and group size (continuous variable) as the response variables. Small groups represent a range from 1 – 6 individuals, medium groups 7 – 12 individuals, and large groups 13 – 18 individuals. For visualization a median group size was selected to predict the curves for each category: 4 (small), 10 (medium), and 16 (large). Shaded regions represent the 95% CI and raw data are plotted as grey shapes for small (circle), medium (plus), and large (inverted triangle). Vertical lines represent the timing of the peak of activity for small (blue) and large groups (red).

Supplemental Table S3: Effects of soil temperature at 40 cm on seasonal activity patterns. Results are from a GLMM using a cosine-sine wave on hourly activity scores (binary, Active = 1, Inactive = 0), including a 3-way interaction between time, year, and temperature. Both hourly time and month were converted to radians. Time-from-Start is the amount of time that passed since the beginning of the reading session, and Temperature is the temperature at 40 cm.

**Predictors**	**Est.**	**S.E.**	**p-value**
(Intercept)	-3.130	0.733	**< 0.001**
sin(hour)	-2.465	0.618	**< 0.001**
cos(hour)	0.752	0.634	0.235
sin(year)	-0.700	0.582	0.229
cos(year)	-0.948	0.743	0.202
Temperature	0.069	0.030	**0.021**
Time-from-Start	-0.012	0.001	**< 0.001**
sin(hour)*sin(year)	0.100	0.266	0.706
sin(hour)*cos(year)	-0.923	0.373	**0.013**
cos(hour)*cos(year)	1.640	0.383	**< 0.001**
cos(hour)*sin(year)	-0.377	0.275	0.171
sin(hour)*Temperature	0.092	0.026	**< 0.001**
cos(hour)*Temperature	-0.053	0.027	**0.047**
sin(year)*Temperature	0.024	0.023	0.296
cos(year)*Temperature	0.011	0.027	0.688
sin(hour)*sin(year)*Temperature	-0.004	0.011	0.737
sin(hour)*cos(year)*Temperature	0.010	0.012	0.391
cos(hour)*cos(year)*Temperature	-0.048	0.012	**< 0.001**
cos(hour)*sin(year)*Temperature	0.016	0.012	0.173

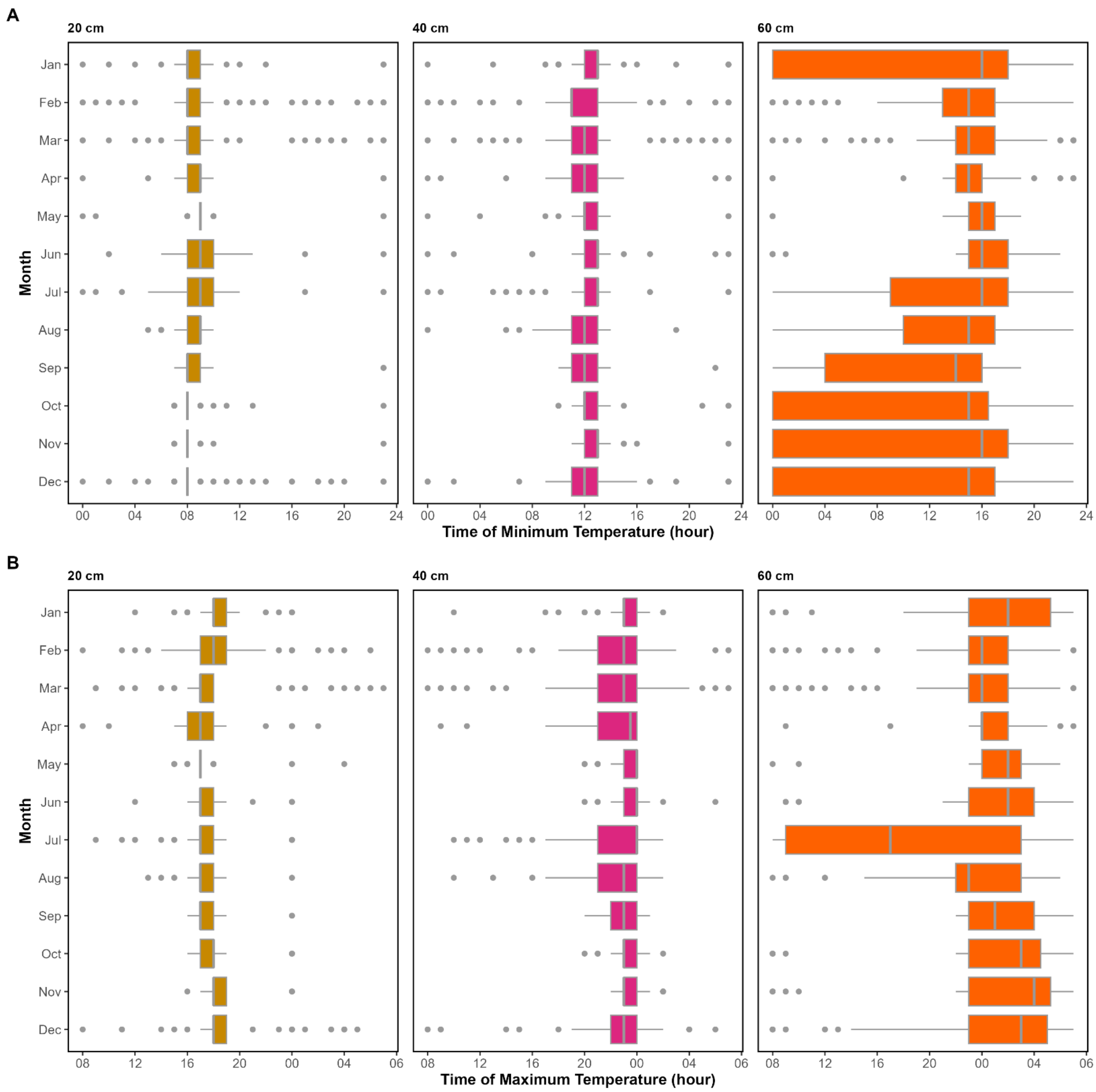

Supplementary Figure S4: Time of A) minimum daily temperature and B) maximum daily temperature at different depths. Boxplots show the distribution of the time of daily minimum and maximum temperature for each month. Dots represent outliers. Note the adjusted x-axis for the lower panels to allow plotting across midnight.

Supplementary Figure S5 (below): Change in diel activity across seasons shown in Figure 2 is here enlarged to two pages to show the variation in temperature at 40 cm below ground.

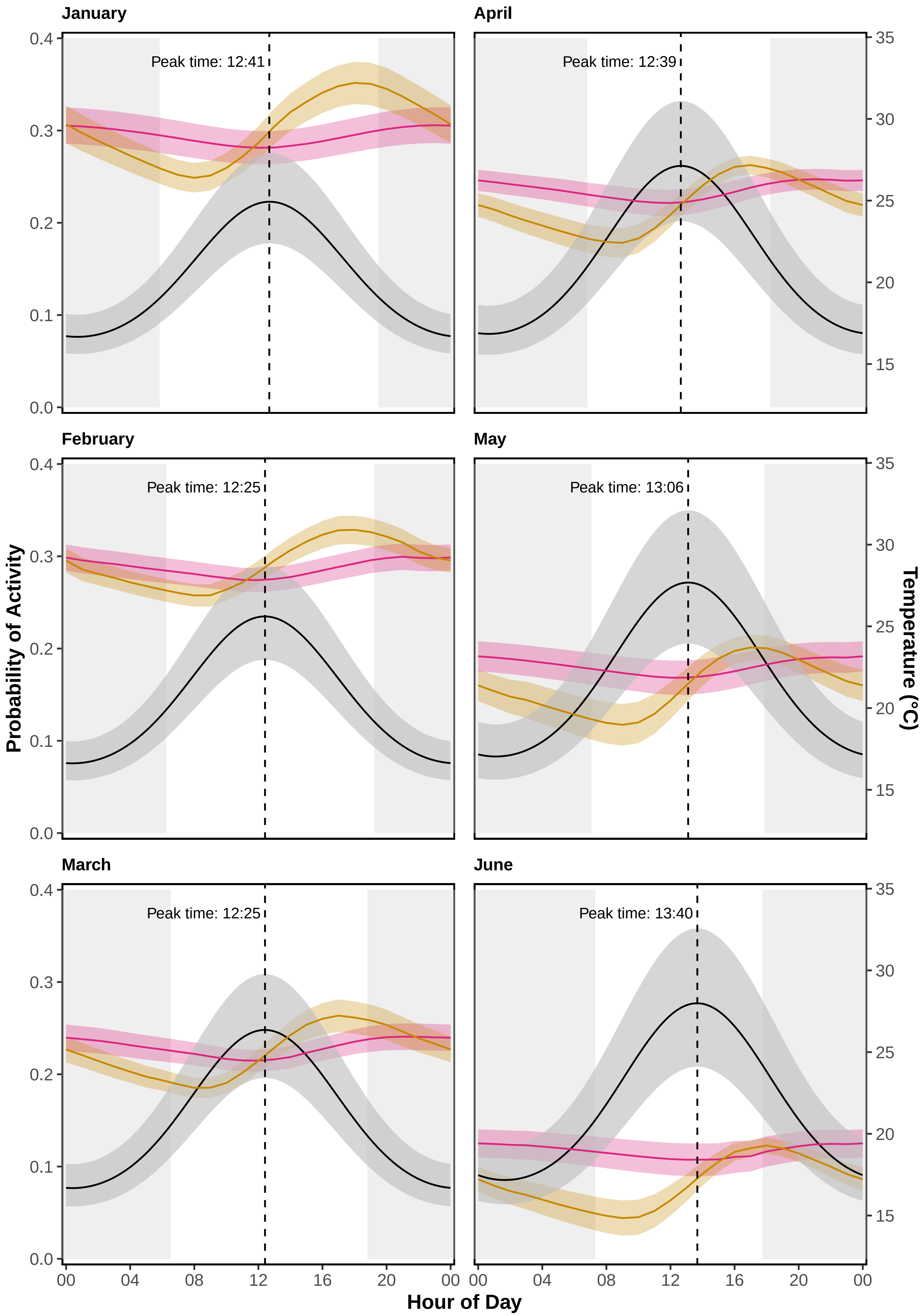

January
Peak time: 12:41
April
Peak time: 12:39
February
Peak time: 12:25
May
Peak time: 13:06
March
Peak time: 12:25
June
Peak time: 13:40
Probability of Activity
Temperature (°C)
Hour of Day
0.0
0.1
0.2
0.3
0.4
15
20
25
30
35
00
04
08
12
16
20
00

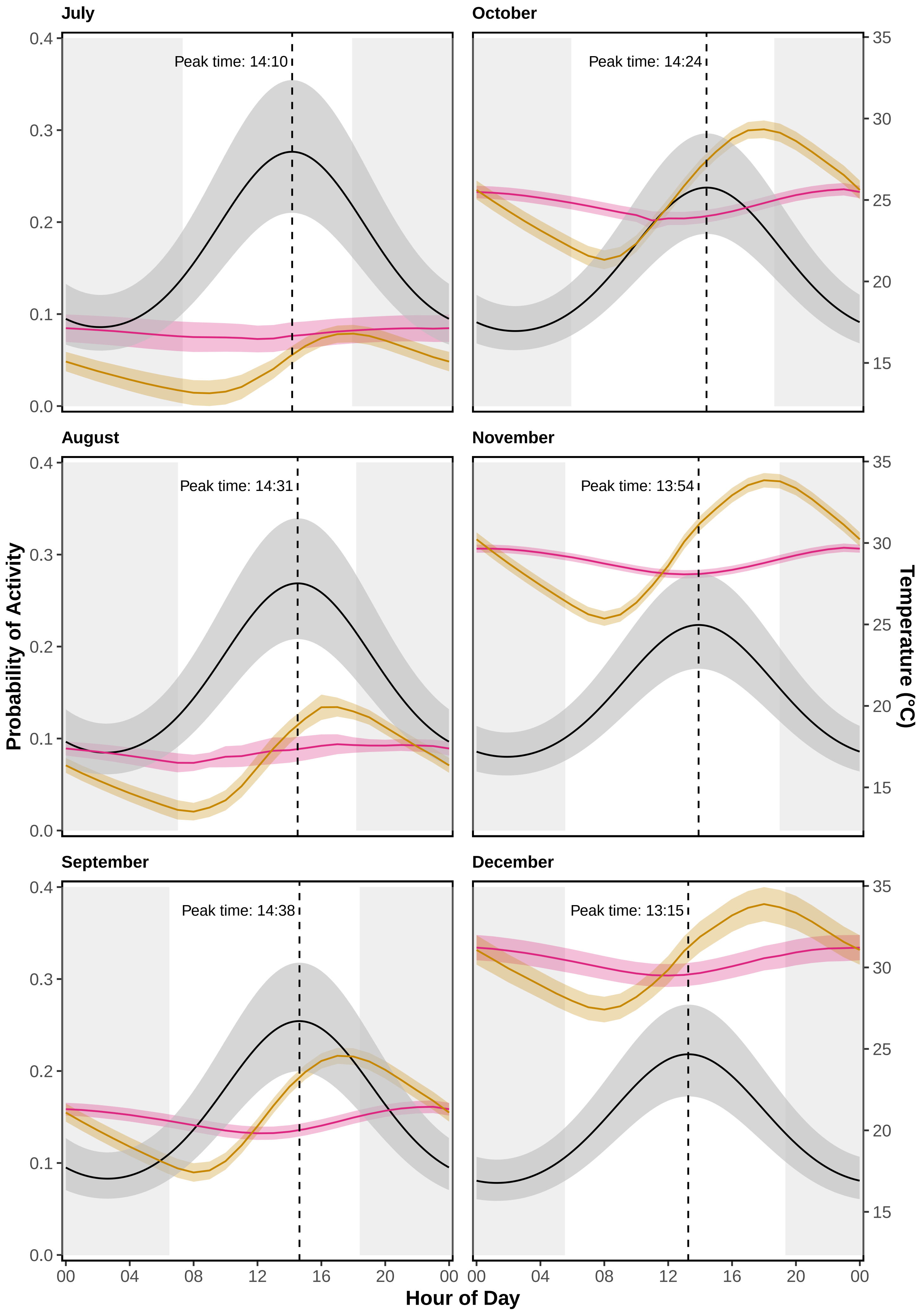
July
October
August
November
September
December
Peak time: 14:10
Peak time: 14:24
Peak time: 14:31
Peak time: 13:54
Peak time: 14:38
Peak time: 13:15
0.0
0.1
0.2
0.3
0.4
15
20
25
30
35
00
04
08
12
16
20
00
Probability of Activity
Temperature (°C)
Hour of Day

Supplemental Table S4: Variation in daily air and soil temperatures for each month at the study site from 2014 – 2019. Values were calculated as the difference in maximum and minimum daily temperature per month and presented as mean ± SD. Air temperature was measured 2 cm above the sand.

	**January**	**February**	**March**	**April**	**May**	**June**
**Air**	25.10 ± 5.85	20.97 ± 5.50	23.32 ± 5.49	22.93 ± 6.34	27.40 ± 5.58	28.56 ± 6.98
**20 cm**	6.22 ± 3.07	5.85 ± 2.79	5.67 ± 2.79	5.43 ± 2.82	4.94 ± 2.87	4.51 ± 2.94
**40 cm**	1.76 ± 1.06	2.30 ± 1.23	2.06 ± 1.07	2.16 ± 1.38	1.53 ± 0.93	1.26 ± 1.02
**60 cm**	0.53 ± 0.47	0.72 ± 0.52	0.69 ± 0.51	0.94 ± 1.31	0.50 ± 0.25	0.44 ± 0.72

	**July**	**August**	**September**	**October**	**November**	**December**
**Air**	25.22 ± 12.46	27.73 ± 8.83	28.60 ± 5.74	28.42 ± 5.78	29.28 ± 5.13	26.33 ± 5.39
**20 cm**	4.23 ± 3.46	7.16 ± 5.16	7.39 ± 1.82	8.30 ± 1.97	8.75 ± 1.91	7.25 ± 3.02
**40 cm**	1.61 ± 2.23	2.89 ± 5.11	1.82 ± 0.40	2.22 ± 2.14	1.90 ± 0.49	2.25 ± 1.55
**60 cm**	1.02 ± 2.16	1.98 ± 5.93	0.52 ± 0.19	0.67 ± 2.01	0.46 ± 0.19	0.56 ± 0.94

Supplemental Table S5: Mean monthly air and soil temperatures at the study site from 2014 – 2019. Values are mean ± SD. Air temperature was measured 2 cm above the sand.

	**January**	**February**	**March**	**April**	**May**	**June**
**Air**	32.2 ± 9.49	31.0 ± 7.84	26.4 ± 8.93	22.8 ± 8.34	17.6 ± 9.91	14.2 ± 10.67
**20 cm**	29.3 ± 6.61	29.8 ± 4.72	25.0 ± 5.17	24.7 ± 4.39	21.2 ±5.30	17. 0 ± 3.90
**40 cm**	29.0 ± 6.05	28.6 ± 4.66	25.3 ± 4.66	25.6 ± 3.95	22.5 ± 4.83	19.0 ± 4.09
**60 cm**	28.6 ± 5.38	28.4 ± 4.57	25.4 ± 4.56	25.7 ± 3.59	22.9 ± 3.88	20.5 ± 4.70

	**July**	**August**	**September**	**October**	**November**	**December**
**Air**	12.8 ± 10.13	16.6 ± 10.40	21.4 ± 10.59	25.5 ± 10.71	29.1 ± 10.93	32.8 ± 9.60
**20 cm**	14.9 ± 3.16	16.5 ± 3.53	21.0 ± 3.50	25.4 ± 4.08	29.7 ± 3.75	30.7 ± 5.84
**40 cm**	16.8 ± 3.84	17.2 ± 2.74	20.7 ± 1.97	24.8 ± 2.31	29.0 ± 1.50	30.4 ± 4.48
**60 cm**	18.5 ± 5.21	17.6 ± 3.36	20.2 ± 1.58	23.9 ± 1.82	27.9 ± 0.99	29.5 ± 3.80
